# Maternal Metabolic Demands Caused by Pregnancy and Lactation: Association with Productivity and Offspring Phenotype in High-Yielding Dairy Ewes

**DOI:** 10.3390/ani9060295

**Published:** 2019-05-30

**Authors:** José Luis Pesántez-Pacheco, Ana Heras-Molina, Laura Torres-Rovira, María Victoria Sanz-Fernández, Consolación García-Contreras, Marta Vázquez-Gómez, Pablo Feyjoo, Elisa Cáceres, Millán Frías-Mateo, Fernando Hernández, Paula Martínez-Ros, Juan Vicente González-Martin, Antonio González-Bulnes, Susana Astiz

**Affiliations:** 1 School of Veterinary Medicine and Zootechnics, Faculty of Agricultural Sciences, University of Cuenca, Avda. Doce de Octubre, Cuenca 010220, Ecuador; jose.pesantez@ucuenca.edu.ec; 2 Department of Animal Reproduction, Instituto Nacional de Investigación Agrarias y Alimentarias (INIA), Avda Pta. de Hierro s/n, 28040 Madrid, Spain; andelash@ucm.es (A.H.-M.); torrerovi@gmail.com (L.T.-R.); mvsanzfernandez@gmail.com (M.V.S.-F.); garcia.consolacion@inia.es (C.G.-C.); bulnes@inia.es (A.G.-B.); 3 Faculty of Veterinary Medicine, Complutense University of Madrid (UCM), Avda. Pta. de Hierro s/n, 28040 Madrid, Spain; mvgomez@ucm.es (M.V.-G.); feyjoo96@gmail.com (P.F.); elicacer@ucm.es (E.C.); millanfr@ucm.es (M.F.-M.); juanvi@vet.ucm.es (J.V.G.-M.); 4 Technical Department, Granja Cerromonte SL, San Juan de la Encinilla, 05358 Ávila, Spain; granja@cerromonte.es; 5 Departamento Producción y Sanidad Animal, Salud Pública Veterinaria y Ciencia y Tecnología de los Alimentos (PASAPTA), Facultad de Veterinaria, Universidad Cardenal Herrera-CEU, Tirant lo Blanc, 7. 46115 Alfara del Patriarca Valencia, Spain; paula.martinez@uchceu.es; 6 Technical Department, TRIALVET SL, C/Encina 22, Cabanillas de la Sierra, 28721 Madrid, Spain

**Keywords:** milk yield, dairy sheep, pregnancy rank, age, metabolic profile, birth weight, sex lamb

## Abstract

**Simple Summary:**

This study assessed the effects of metabolic load imposed by pregnancy and lactation on productivity and offspring performance in high-yielding dairy sheep. Productivity was assessed in terms of offspring and maternal milk yield, metabolic profile, and body condition. Our results show that maternal productivity and lamb body weight and growth are not compromised by pregnancy and lactation because dairy sheep, when appropriately managed, seem to be able to cover metabolic demands of pregnancy and high milk production without losing productivity.

**Abstract:**

Pregnancy and lactation, especially when concurrent, create a rather metabolically demanding situation in dairy ruminants, but little is known about their effects on offspring phenotype and milk yield. Here, we evaluated the impact of pregnancy and lactation on the metabolic traits and productive performance of Lacaune dairy sheep and their offspring. Productive performance was measured in terms of milk yield, body weight (BW), body condition score (BCS), and size. Productivity was assessed during mid-pregnancy (75 ± 5 d) and late pregnancy (142 ± 4 d) and at 52 ± 5 d in the postpartum period. During pregnancy, high-yielding ewes had higher BW, BCS, plasma glucose, cholesterol, β-OHB, and NEFA than low-yielding ewes, but lower levels of lactate and urea. High-yielding animals had lower BCS after lambing, but their lambs showed greater growth. Productivity during lactation was affected by ewe age and parity: Mature ewes (but not maiden sheep) whose BCS increased steeply during pregnancy yielded more milk in the subsequent lactation than those whose BCS did not increase. Lamb BW and size were positively associated with milk yield in the subsequent lactation. Mature ewes had higher yields than maiden sheep, and mature ewes with multiple pregnancies produced more milk than those with singleton pregnancies. Ewes with male singleton pregnancies also showed higher yield than those with female singletons. These results demonstrate that high-yielding dairy sheep, when appropriately fed and managed, can adequately cover the metabolic demands of pregnancy and high milk production (even when concurrent) without losing productivity.

## 1. Introduction

Pregnancy and lactation are associated with major metabolic changes in high-yielding dairy ruminants due to the increased requirement for nutrients such as glucose. In pregnant but non-lactating ewes, the uterus and placenta are the most energetically-demanding tissues until mid-pregnancy. Placental energy consumption decreases over the course of pregnancy, and by the end of gestation, most energy is transferred to the fetus [1]. Concurrent pregnancy and lactation are even more energetically demanding. Lactation requires dramatic metabolic changes in mammary and nonmammary tissues [1]. The main nutrient consumed is glucose [1,2], which is required for the synthesis of lactose, the most important osmotic solute in milk [3]. In high-yielding dairy cattle, the mammary requirements of glucose a few days after calving are more than 2.7 times greater than that of the gravid uterus during late pregnancy [1]. In dairy goats, mammary glucose uptake becomes even greater than oxygen consumption or blood flow immediately before parturition in order to meet the requirements of milk production [2].

The high metabolic demands of pregnancy and lactation must be supported by increased uptake of nutrients. However, dairy ruminants and especially dairy ewes cannot sufficiently increase dry matter intake during early lactation (postpartum) and late pregnancy because much of the available space is or was (postpartum) already occupied by the gravid uterus [4]. These stages also coincide with the greatest fetal energy demand (late pregnancy) and an increase in milk yield (postpartum). As a result, ewes are in a state of negative energy balance during early lactation and late pregnancy.

To cope with the challenge of negative energy balance during pregnancy and lactation, ewes increase their diurnal feeding frequency, decrease lipogenic activity, and increase lipolysis and proteolysis in order to mobilize endogenous substrates stored in body reserves [1,3,5,6,7]. Few studies have investigated whether these strategies are sufficient to maintain productivity and health in intensive dairy production systems. The severity of the metabolic challenge and dietary adjustment required to compensate can be evaluated by measuring ewe body weight (BW), body condition score (BCS), and blood metabolites [8,9]. 

The metabolic condition of the ewe is also an important determinant of offspring development and birth weight. Birth weight is determined by both genetic and environmental factors, such as maternal nutrition, metabolism, and placental function [10,11]. Maternal metabolic challenges may therefore compromise the health and future productivity of offspring. Competition between maternal and fetal needs can affect maternal health and offspring birth weight [12], especially in young ewes that need to support their own growth in addition to pregnancy requirements [12]. When pregnancy and lactation are concurrent, the fetus must compete with the demands of lactation for nutrients [13,14]. Previous studies have demonstrated a negative correlation between high milk yield and birth weight in dairy cattle [13,15] with an interaction between maternal age and number of lactations of the cow. High-yielding cows with a parity of three or more are also more likely to give birth to calves with low birth weight [15].

The metabolic challenge of pregnancy, with or without concurrent lactation, may also decrease reproductive and productive efficiency in the mother [16]. The most energetically demanding periods (late pregnancy and early lactation) can even cause fatty liver disease, pregnancy toxaemia, and ketosis, thereby affecting milk production [16,17]. Ewe body weight and size, BCS, milk production, offspring sex, and even seasons all influence how well sheep adapt to the metabolic stress of pregnancy and lactation [16,18,19,20,21]. Understanding these factors is key for optimizing and maintaining sustainable dairy production systems. However, few studies have investigated the effects of pregnancy and lactation on intensively managed dairy ewes. 

The aim of the present study was to assess the metabolic demands during pregnancy and concurrent lactation in high-producing Lacaune dairy sheep and examine the subsequent effects on maternal milk yield, offspring metabolism and body size. This work is closely related to another study in this same Special Issue [22], in which we assessed the effects of maternal body weight, condition, parity, and pregnancy rank on metabolism in ewes and their offspring.

## 2. Materials and Methods

**Figure 1:**
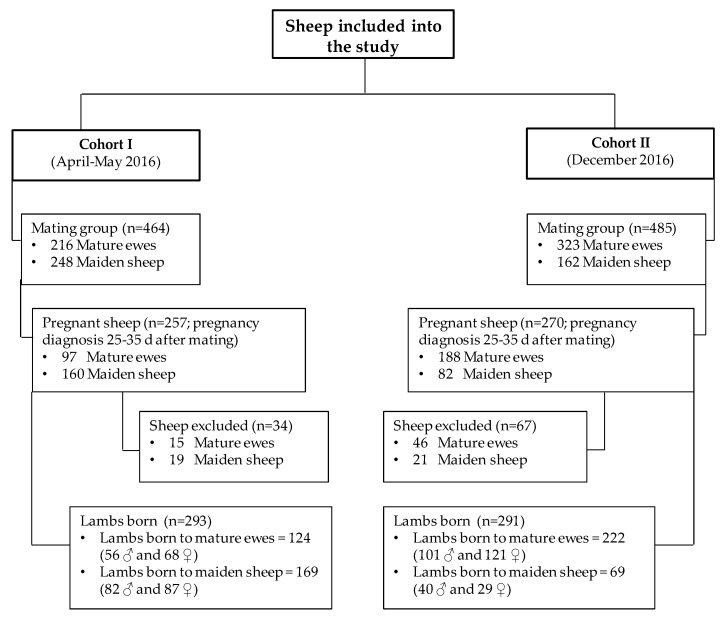
Flow chart of sheep included in the study.

This work was carried out concurrently with another study published in the same Special Issue of this Journal as two parts of the same project, using the same flock [22]. The study design, animals used, methods for animal handling, measurements of metabolic parameters in ewes and lambs, assessments of postnatal development, and statistical analyses were all performed as described in that work [22]. Altogether, the study included 527 pregnant ewes (285 mature and 242 maiden), from which 34 were lost from the first mating group and 67 lost from the second group during late pregnancy or parturition because of death (8 ewes), abortion (3), non-pregnancy (7), lambings after the date predicted (3), or an absence of identification of lambs born (80). Subsequently, a total of 584 lambs (305 females and 279 males) were included in the study, of which 346 were born to mature ewes and 238 to maiden ewes. A global lamb mortality of 5.82% was observed (34/584 lambs), and 550 lambs could be measured and sampled at the age of 17 days. Ewes without previous offspring were defined as maiden sheep, while those with a parity of 1 or more were defined as mature ewes. A summary of the two cohorts of ewes in this study is shown in Figure 1.

### 2.1. Assessment of Maternal Milk Yield

**Figure 2:**
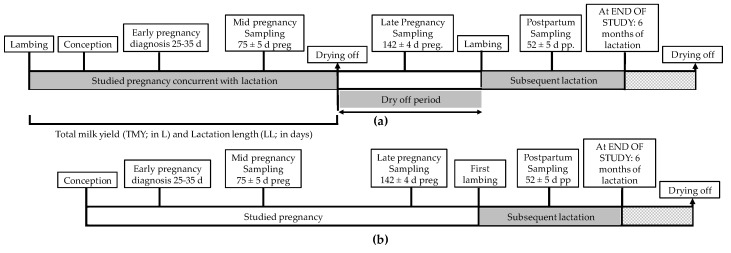
Timeline of assessments in mature ewes (**a**) and maiden sheep (**b**).

Maternal milk yield during pregnancy was assessed in mature ewes. Milk yield was measured at mid-pregnancy, late-pregnancy, and postpartum (Figure 2a). Parameters measured included:

- Total milk yield over the lactation period (TMY). Based on the own data and percentile distributions, ewes with a TMY of <220 L were defined as low-yielding (L-TMY); those with 220 –371 L as average-yielding (A-TMY); and those with >371 L as high-yielding (H-TMY).
- Yield per day in milk (YDIM) from conception to drying off (YDIMd). Ewes with a YDIMd of <0.77 L/d were defined as low-yielding (L-YDIMd); those with 0.77–1.12 L/d as average-yielding (A-YDIMd); and those with >1.12 L/d as high-yielding (H-YDIMd).
- YDIM during the month of conception (YDIMc). Ewes with a YDIMc of <0.91 L/d were defined as low-yielding (L-YDIMc); those with 0.91–1.35 L/d as average-yielding (A-YDIMc); and those with >1.35 L/d as high-yielding (H-YDIMc).

Maternal milk yield was also assessed during the first 6 months after lambing in both mature ewes and maiden sheep (subsequent lactation, Figure 2a,b). Ewes with a YDIM after lambing (YDIMs) of <1.57 L/d were defined as low-yielding (L-YDIMs); those with 1.57–2.19 L/d as average-yielding (A-YDIMs); and those with >2.19 L/d as high-yielding (H-YDIMs).

### 2.2. Assessment of Productivity

Data on productivity were collected from mature ewes for lactation during pregnancy (concurrent pregnancy and lactation) and from the lactation after lambing for both maiden sheep and mature ewes (subsequent lactation, Figure 2). Productivity parameters were measured at mid-pregnancy, late-pregnancy, and postpartum.

The productivity parameters assessed for concurrent pregnancy and lactation included the date of lambing; lactation length (LL); total milk yield (TMY); dry period length (DPL); and YDIM during the entire lactation (YDIMt), before conception (YDIMb), during the month of conception (YDIMc), during the second and third months of pregnancy, or from conception to drying off (YDIMd).

The productivity parameters assessed for the subsequent lactation included the date of lambing, lactation length (LLs), total milk yield (TMYs), YDIM during the first 6 months after lambing (YDIMs) and during the whole lactation period (YDIMts), and dry period length (DPLs). 

### 2.3. Statistical Analyses

Data were analyzed using SPSS^®^ 22.0 (IBM, Armonk, NY, USA) by the Statistical Department of the Center for Research Support of Complutense University of Madrid, Spain. Changes over time in BW, BCS, BMI, length, and metabolic indices of sheep and lambs were assessed for significance using ANOVA for repeated measures in a general linearized model with Greenhouse-Geisser correction. Differences between groups at different time points were assessed for significance using non-parametric analysis, the Kruskal-Wallis test or the Mann-Whitney test, after confirming the skewed (non-normal) distribution of the data. Inter-group differences in body circumference, average daily gain and metabolic indices in lambs were also assessed for significance using the independent *t* test. Pearson correlation coefficients were calculated to assess the strength of potential relationships between sheep variables, or of relationships of lamb birth weight with maternal metabolic indices. Data were expressed as mean ± S.E.M., and differences were considered significant if *p* < 0.05. Differences associated with *p* between 0.05 and 0.09 were defined as tendencies.

## 3. Results

### 3.1. Productivity

Productivity in ewes during concurrent lactation and gestation and in the subsequent lactation alone are summarized in Table 1.

Classification of ewes based on the level of milk yield during concurrent gestation and lactation (mature ewes only) and during the subsequent lactation (mature ewes and maiden sheep) is shown in Table 2. 

### 3.2. Maternal Body Weight and Condition Correlated with Milk Yield in Mature Ewes

**Figure 3:**
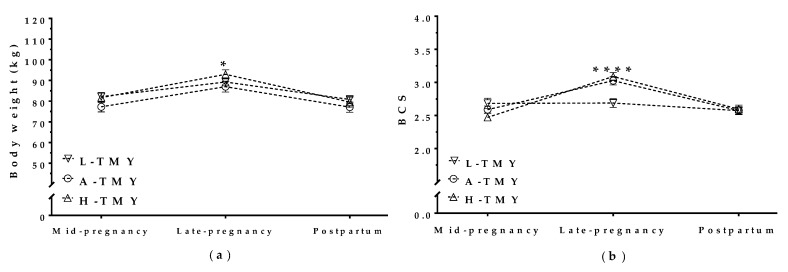
Maternal body weight (**a**) and body condition score (**b**) in mature ewes with high, average, and low total milk yield during concurrent gestation and lactation and in the postpartum period; L-TMY: low-yielding sheep with a TMY (total Milk Yield) <220 L/lactation; A-TMY: average yielding sheep (220–371 L); H-TMY: high-yielding sheep (>371 L). Data are mean ± standard error of the mean (SEM). Significant interactions between pregnancy/postpartum stage and TMY (*p* < 0.05 for BW and *p* < 0.001 for BCS). Asterisks indicate significant differences between groups at each time point (**** *p <* 0.0001; * *p* < 0.05).

TMY correlated with BW and BCS in mature ewes during concurrent gestation and lactation and interacted significantly with the pregnancy/postpartum stage. High-yielding mature ewes (H-TMY) had higher BW in late pregnancy than average-yielding mature ewes (A-TMY; *p* = 0.03), but there was no difference in BW between H-TMY ewes and L-TMY ewes (Figure 3a). H-TMY and A-TMY mature ewes also had higher BCS in late pregnancy than L-TMY ewes (*p* < 0.001). The BCS of L-TMY ewes did not change throughout pregnancy or after lambing (Figure 3b).

In contrast, YDIMd and YDIMc did not correlate with maternal BW (*p* = 0.7 for YDIMd and *p* = 0.1 for YDIMc) or BCS (*p* = 0.1 for YDIMd and *p* = 0.2 for YDIMc) during concurrent pregnancy and lactation or in the postpartum period. 

### 3.3. Maternal Metabolite Levels Correlated with Milk Yield in Mature Ewes

**Figure 4:**
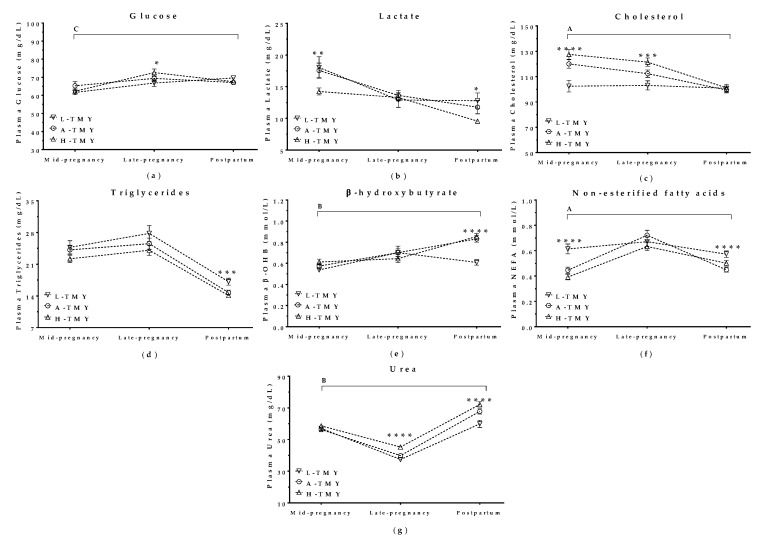
Plasma glucose (**a**), lactate (**b**), cholesterol (**c**), triglycerides (**d**), β-hydroxybutyrate (**e**), non-esterified fatty acids (**f**), and urea (**g**) in mature ewes with high, average, or low total milk yield during concurrent gestation and lactation and in the postpartum period. L-TMY: low-yielding sheep with a TMY (total Milk Yield) <220 L/lactation; A-TMY: average yielding sheep (220–371 L); H-TMY: high-yielding sheep (>371 L). Data are mean ± SEM. Significant interactions between pregnancy/postpartum stage and TMY are represented by an upper horizontal line and capital letters (A: *p* < 0.0001, B: *p* < 0.001, and C: *p* < 0.05). Asterisks indicate significant differences between groups at each time point (**** *p <* 0.0001; *** *p* < 0.001; ** *p* < 0.01; * *p* < 0.05).

Plasma metabolite levels correlated with TMY in mature ewes both during concurrent gestation and lactation and during the subsequent lactation after lambing. Glucose, cholesterol, and urea levels were all higher in H-TMY ewes than in L-TMY ewes, and there was an interaction with the pregnancy/postpartum stage (Figure 4a,c,g). Lactate and triglyceride levels decreased throughout pregnancy and the postpartum period independently of TMY (Figure 4b,d). β-hydroxybutyrate (β-OHB) levels increased during pregnancy in all ewes but decreased after lambing only in L-TMY ewes (*p* < 0.001, Figure 4e). Finally, non-esterified fatty acids increased during pregnancy and decreased after lambing in all ewes, but were higher in L-TMY ewes than H-TMY and A-TMY ewes during mid-pregnancy and the postpartum period (Figure 4f). 

There was no correlation between YDIMd (*p* > 0.05) or YDIMc (*p >* 0.05) and maternal metabolite levels during concurrent pregnancy and lactation or in the postpartum period.

### 3.4. Lamb BW, Body Size, and Plasma Metabolites Correlated with Maternal Milk Yield in Mature Ewes

Birth weight, trunk length, body mass index (BMI), and plasma metabolites in lambs born to mature ewes are shown in Table 3. There was no difference in birth weight among lambs born to high-, average- or low-yielding ewes. However, lambs born to high-yielding (H-TMY) ewes showed higher average daily weight gain than those born to average- or low-yielding ewes (*p* < 0.05). At 17 days of age, lambs born to high-yielding ewes were heavier (*p* < 0.0001) and had greater trunk length, BMI, and thoracic and abdominal girth than lambs born to low-yielding ewes. Birth weight (*p* = 0.006), but not trunk length, correlated with YDIMd, while both birth weight and trunk length positively correlated with YDIMc. There was no correlation between YDIMc and lamb phenotypes at 17 days of age (Table 3).

Plasma β-OHB and urea levels were higher in lambs born to H-TMY ewes than in those born to A-TMY and L-TMY ewes (*p* < 0.005 for both), while lactate levels were lower in lambs born to high-yielding than low-yielding ewes (*p* < 0.0001). Plasma β-OHB levels were also higher (*p* < 0.05), and lactate levels lower (*p* < 0.01), in lambs born to H-YDIMd ewes than in those born to L-YDIMd ewes, but there was no correlation with YDIMc. Lambs born to L-YDIMc ewes also had lower levels of glucose than those born to A-YDIMc or H-YDIMc ewes at 17 days of age (Table 3).

Details of Pearson correlation analysis between milk yield during gestation and lamb birth weight are shown in Table S1. A weak, positive correlation was found between high-yielding ewes and lamb birth weight (*r* < 0.34, *p* < 0.05), with a stronger effect in fatter mothers and in ewes with high YDIMc instead of high YDIMd. Lamb birth weight also positively correlated with YDIMc in mature ewes with multiple pregnancies. 

Lamb birth weight showed a weak positive correlation with the following maternal levels during mid-pregnancy: glucose (*r* = 0.17, *p* < 0.05), lactate (*r* = 0.24, *p* < 0.05), cholesterol (*r* = 0.18, *p* < 0.05), triglyceride (*r* = 0.24, *p* < 0.05), and NEFA (*r* = 0.23, *p* < 0.01). In contrast, maternal β-OHB and NEFA levels in late-pregnancy showed a weak negative correlation with lamb birth weight (all *r* values < −0.25, *p* < 0.05; Table S2).

### 3.5. Maternal Features during Gestation on Milk Yield during the Subsequent Lactation

#### 3.5.1. Maternal Weight and BCS during Concurrent Gestation and Lactation Affected Milk Yield in the Subsequent Lactation

**Figure 5:**
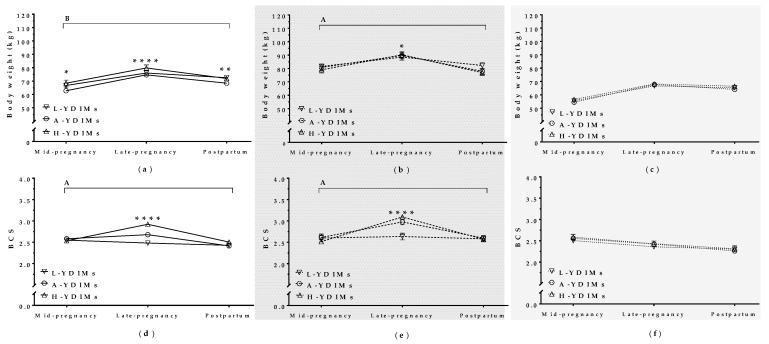
Maternal body weight in (**a**) all ewes, (**b**) mature ewes, and (**c**) maiden sheep showing high, average, or low milk yield at different stages of pregnancy and the postpartum period. BCS in (**d**) all ewes, (**e**) mature ewes, and (**f**) maiden sheep showing high, average, or low milk yield at different stages of pregnancy and the postpartum period. L-YDIMs: low-yielding sheep with a YDIMs (daily milk yield during the first 6 months after lambing) of 1.17 L/d in average; A- YDIMs: average yielding sheep with a YDIMs of 1.90 L/d in average; H-YDIMs: high yielding sheep with a YDIMs of 2.70 L/d in average. Data are mean ± SEM. Significant interactions between pregnancy/postpartum stage and YDIMs are represented by an upper horizontal line (A: *p* < 0.0001; B: *p* < 0.01). Asterisks indicate significant differences between groups at each time point (**** *p <* 0.0001; ** *p* < 0.01; * *p* < 0.05).

To investigate how metabolic challenge during a previous pregnancy (with or without concurrent lactation) affected productivity in the subsequent lactation after lambing, we compared milk yield after lambing (YDIMs) in mature ewes and maiden sheep (Figure 5). Mature ewes with high YDIMs also showed higher increases in maternal BW (*p* = 0.01) and BCS (*p* < 0.0001) during pregnancy, particularly in late pregnancy (Figure 5b, e). This effect was not observed in maiden sheep, where YDIMs was independent of BW and BCS during pregnancy (Figure 5c,f).

#### 3.5.2. Maternal Metabolite Levels during Pregnancy Correlated with Productivity in the Subsequent Lactation

##### Glucose

**Figure 6:**
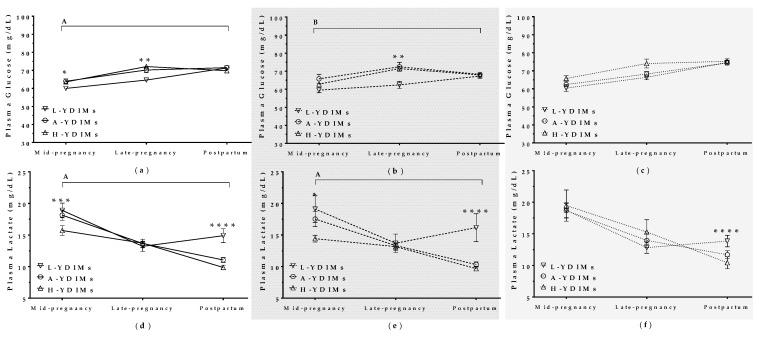
Plasma glucose levels in (**a**) all ewes, (**b**) mature ewes, and (**c**) maiden sheep showing high, average, or low milk yield at different stages of pregnancy and the postpartum period. Lactate levels in (**d**) all ewes, (**e**) mature ewes, and (**f**) maiden sheep showing high, average, or low milk yield at different stages of pregnancy and the postpartum period. L-YDIM: low-yielding sheep with a TMY (total Milk Yield) <220 L/lactation; A-TMY: average yielding sheep (220–371 L); H-TMY: high-yielding sheep (>371 L). Data are mean ± SEM. Significant interactions between pregnancy/postpartum stage and YDIMs are represented by an upper horizontal line (A: *p* < 0.0001; B: *p* < 0.05). Asterisks indicate significant differences between groups at each time point (**** *p* < 0.0001; *** *p* < 0.001; ** *p* < 0.01; * *p* < 0.05).

Mature H-YDIMs and A-YDIMs ewes had higher glucose levels in late pregnancy than L-YDIMs ewes (*p* < 0.0001, Figure 6b). However, there was no difference in glucose levels between H-YDIMs and L-YDIMs maiden sheep (Figure 6c).

##### Lactate

L-YDIMs ewes and maiden sheep showed higher plasma lactate levels in the postpartum period than H-YDIMs and A-YDIMs ewes and maiden sheep. The difference was larger for mature ewes (Figure 6e,f).

##### Cholesterol

**Figure 7:**
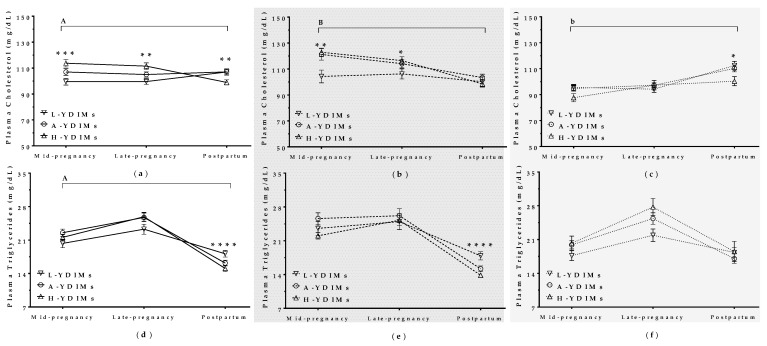
Plasma cholesterol in (**a**) all ewes, (**b**) mature ewes, and (**c**) maiden sheep showing high, average, or low milk yield at different stages of pregnancy and the postpartum period. Triglycerides in (**d**) all ewes, (**e**) mature ewes, and (**f**) maiden sheep showing high, average, or low milk yield at different stages of pregnancy and the postpartum period. L-YDIMs: low-yielding sheep with a YDIMs (daily milk yield during the first 6 months after lambing) of 1.17 L/d in average; A- YDIMs: average yielding sheep with a YDIMs of 1.90 L/d in average; H-YDIMs: high yielding sheep with a YDIMs of 2.70 L/d in average. Data are mean ± SEM. Significant interactions between pregnancy/postpartum stage and YDIMs are represented by an upper horizontal line (A: *p* < 0.0001; B: *p* < 0.001; and b: *p* < 0.06). Asterisks indicate significant differences between groups at each time point (**** *p* < 0.0001; *** *p* < 0.001; ** *p* <0.01; * *p* < 0.05).

The relationship between milk yield and cholesterol was different for mature ewes than for maiden sheep (Figure 7). Mature H-YDIMs and A-YDIMs ewes had higher cholesterol levels than L-YDIMs ewes in mid-pregnancy, and these levels then decreased after lambing (Figure 7b). Mature L-YDIMs ewes had low cholesterol levels throughout gestation and the postpartum period. In contrast, H-YDIMs and A-YDIMs maiden sheep had higher cholesterol levels than L-YDIMs sheep in the postpartum period (Figure 7c). L-YDIMs maiden sheep showed an increase in cholesterol during gestation and after lambing. 

##### Triglycerides

Triglyceride levels remained stable during gestation and decreased after lambing in all sheep, but were lower in mature H-YDIMs and A-YDIMs ewes than L-YDIMs ewes in the postpartum period (Figure 7e). There was no difference in triglyceride levels among H-YDIMs, A-YDIMs, and L-YDIMs maiden sheep (Figure 7f).

##### β-hydroxybutyrate (β-OHB)

**Figure 8:**
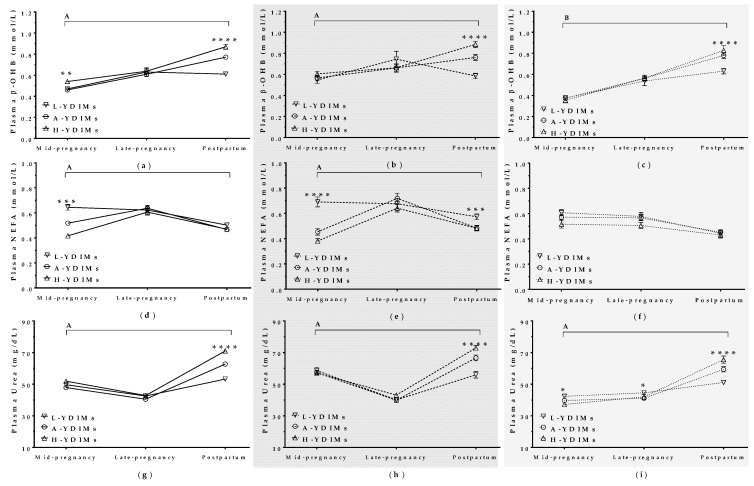
Plasma β-hydroxybutyrate levels in (**a**) all ewes, (**b**) mature ewes, and (**c**) maiden sheep showing high, average, or low milk yield at different stages of pregnancy and the postpartum period. Non-esterified fatty acids (NEFA) in (**d**) all ewes, (**b**) mature ewes, and (**f**) maiden sheep showing high, average, or low milk yield at different stages of pregnancy and the postpartum period. Urea in (**g**) all ewes, (**h**) mature ewes, and (**i**) maiden sheep showing high, average, or low milk yield at different stages of pregnancy and the postpartum period. L-YDIMs: low-yielding sheep with a YDIMs (daily milk yield during the first 6 months after lambing) of 1.17 L/d in average; A- YDIMs: average yielding sheep with a YDIMs of 1.90 L/d in average; H-YDIMs: high yielding sheep with a YDIMs of 2.70 L/d in average. Data are mean ± SEM. Significant interactions between stage of pregnancy/postpartum and YDIMs are represented by an upper horizontal line (A: *p* < 0.0001; B: *p* < 0.001) Asterisks indicate significant differences between groups at each time point (**** *p* < 0.0001; *** *p* < 0.001; ** *p* < 0.01; * *p* < 0.05).

There was no difference in plasma β-OHB levels in mature ewes until the postpartum period, when mature L-YDIMs ewes showed an abrupt drop in β-OHB levels, whereas β-OHB levels continued to rise in H-YDIMs and A-YDIMs ewes (Figure 8b). β-OHB increased in maiden sheep throughout pregnancy and the postpartum period, but L-YDIMs maiden sheep showed lower β-OHB levels than H-YDIMs and A-YDIMs sheep in the postpartum period (Figure 8c).

##### Non-Esterified Fatty Acids (NEFA)

There was no difference in plasma NEFA levels among H-YDIMs, A-YDIMs, and L-YDIMs maiden sheep. However, NEFA levels were higher in mature L-YDIMs ewes than H-YDIMs and A-YDIMs ewes in mid-pregnancy and in the postpartum period (Figure 8e). 

##### Urea

Plasma urea levels decreased from mid to late pregnancy in mature ewes and increased after lambing. However, H-YDIMs and A-YDIMs ewes had higher postpartum urea levels than L-YDIMs ewes. Urea levels also increased after lambing in maiden sheep, but were higher in H-YDIMs and A-YDIMs maiden sheep than in L-YDIMs maiden sheep.

#### 3.5.3. Ewe Age, Pregnancy Rank, and Concurrent Lactation with Pregnancy Affected Milk Yield in the Subsequent Lactation

Milk yield (YDIMs and YDIMts) was higher, and LLs shorter, in mature ewes than in maiden sheep (Table 4). DLPs was also longer in mature ewes than in maiden sheep (Table 4; *p* < 0.05 for all). Mature ewes with multiple pregnancies also produced more milk (higher YDIMs and YDIMts) after lambing than mature ewes with singleton pregnancies (*p* = 0.04). This effect was not observed in maiden sheep.

Pearson’s correlation analysis also showed a strong positive correlation in mature ewes between milk yield in the lactation concurrent with pregnancy as well as in the subsequent lactation (*r* = 0.627, *p* < 0.0001; Table S3). 

### 3.6. Lamb Phenotypes Correlated with Maternal Yield during the Subsequent Lactation

Lamb corpulence was positively associated with daily milk yield in the subsequent lactation after lambing, with H-YDIMs ewes producing larger lambs at birth than L-YDIMs ewes (Table 5).

Offspring sex was also associated with milk yield after lambing. Ewes which have lambed male lambs produced more milk after lambing than ewes which had lambed female lambs (Table 6; *p* = 0.04). This effect was not observed in ewes with multiple pregnancies. However, mature ewes with multiple pregnancies in which both lambs were female showed higher milk yield in the subsequent lactation than mature ewes with male and female lambs (Table 7; *p* = 0.02).

## 4. Discussion

Our results show that sheep with higher dairy yields during concurrent gestation and lactation have higher BW and better BCS than those with low yield. Both BW and BCS increased throughout pregnancy in high-yielding ewes, whereas low-yielding sheep did not show this increase. These findings suggest that ewes with more body reserve and a positive energy balance are better able to cope with the metabolic challenge imposed by pregnancy and concurrent lactation.

High-yielding ewes also had higher glucose and cholesterol levels during pregnancy. Glucose is the main nutrient consumed during pregnancy and lactation, as it is the main energy source for developing fetuses [23,24,25] and the main component in lactose synthesis [23]. Cholesterol is a key nutrient in tissue development and a precursor for many hormones and metabolic regulators required for fetal development [24,25]. High plasma cholesterol is a marker of good metabolic condition in lactating female dairy cows [26]. In agreement with our findings, previous studies in Merinolandschaf ewes have reported that high-yielding ewes have higher plasma glucose and cholesterol levels [27]. 

High-yielding ewes also showed a steep decrease in BW and BCS after lambing, which was not observed in low-yielding ewes. This decrease may be a consequence of the intense metabolic challenge imposed by high milk production, which causes a negative energy balance [28,29] or may be that they are better mobilizing fat reserves and nutrients. It is noteworthy that all metabolite levels and all measures of BCS were within the normal physiological ranges, regardless of productivity.

In contrast, low-yielding ewes maintained stable BW and BCS during pregnancy and in the postpartum period, suggesting a weaker metabolic challenge reflecting, probably, their low milk production and the disability to mobilize body reserves. Our study design does not allow us to determine whether low-yielding ewes produce less milk because of a limitation in their metabolic status, or if a low milk yield causes metabolic changes. 

NEFA levels are a marker of lipid catabolism, and they increase when glucose metabolism is deficient. After lambing, a simultaneous decrease in NEFA levels and increase in β-OHB levels signals the end of a negative energy balance and lipomobilization stops [30,31]. In our study, high-yielding ewes had the highest levels of β-OHB and the smallest levels of NEFAs in the postpartum period, suggesting that these changes occurred earlier than in low-yielding ewes despite more milk production. High-yielding ewes are therefore more able to produce milk while maintaining maternal health. These changes are similar to those previously described in heifers and sheep [1,4,32] and reflect the high metabolic challenge imposed by concomitant pregnancy and lactation.

Pathological levels of β-OHB are observed in situations associated with metabolic challenge. Dairy goats with ketonemia show reduced milk production before and after giving birth [33]. Similarly, Chios dairy ewes with high levels of β-OHB and NEFA before and after parturition show lower milk yield during lactation [16]. We did not observe this association in the present study, however, possibly because β-OHB and NEFA levels were always within the physiological ranges, reflecting the absence of a severe metabolic stress, despite high milk production.

Maternal milk yield did not affect BW, body size, or BMI of neonatal lambs. We have previously demonstrated that maternal metabolite levels have a negligible effect on lamb phenotype [22]. These results may suggest that high-yielding sheep are more able to cope with the metabolic challenges of lactation and pregnancy. The lightest lambs were produced by ewes with average milk yield. It may be that average-yielding ewes attempt to produce more milk but are not able to cope with the metabolic stress and give birth to lighter lambs as a result. In contrast, low-yielding ewes have a different metabolite profile, suggesting that they direct all their resources into fetal development rather than milk production. Low birth weight is associated with lower metabolite availability in late pregnancy [1,34]. This is consistent with our observation in the present study that sheep that maintained higher levels of glucose, cholesterol, and triglycerides during mid-pregnancy gave birth to heavier lambs.

High birth weight in lambs correlated with lower maternal levels of β-OHB and NEFAs in late pregnancy, especially in average- and high-yielding ewes. These parameters are indicative of body reserve mobilization. Therefore, while previous studies in Border Leicester X Scottish Blackface and Assaf sheep showed that metabolic challenge in mothers negatively affected lamb birth weight [12,35], the high-yielding ewes in our study were able to cope adequately with the metabolic challenge. In support of this, lambs born to high-yielding ewes were larger than those of low- or average-yielding ewes at 17 days of age, despite being born with a slightly, numerically (not significantly different) lower birth weight [20,36]. 

Plasma metabolite levels in lambs correlate weakly with those in the mother in Lacaune ewes [22], which we also observed in our study. For example, β-OHB and urea levels were higher and lactate levels were lower in lambs born to high-yielding ewes than in those born to low-yielding ewes. Similarly, high-yielding ewes had the highest levels of β-OHB and urea and the lowest levels of lactate. These results are similar to those reported in previous studies in meat sheep breeds [12,37].

Productivity parameters in mature ewes during concurrent gestation and lactation were similar to those during the lactation after lambing. Previous studies in Lacaune ewes have shown that milk yield in the current lactation correlates with milk yield in the next lactation, suggesting that individual sheep tend to maintain the same level of yield throughout life [18,38], similar to dairy cattle [39]. These results indicate that productivity is likely to be a heritable genetic trait in dairy ruminants [39] and that previous yield parameters can be used to accurately design culling schemes in a herd. 

The evaluation of maternal metabolites during pregnancy showed that high-yielding sheep had higher plasma concentrations of glucose, lactate, β-OHB and urea, but lower triglycerides and NEFAs, than low-yielding ewes in late pregnancy and postpartum. These differences suggest that low-yielding sheep have reduced metabolic waste, while high-yielding sheep experience greater metabolic challenge in late pregnancy. It is well-known that the metabolic challenge of pregnancy can decrease reproductive and productive efficiency in the subsequent lactation (especially in concurrent gestation and lactation or in maiden sheep with their own growth demands) [17] and may even lead to metabolic diseases such as fatty liver, pregnancy toxemia and ketosis, which also affect milk production [16,17]. However, milk yield in the subsequent lactation was not reduced in our high-yielding ewes in this study. In fact, we found that ewes with the highest yield during concurrent gestation and lactation had the highest BCS and BW during pregnancy and also had high yields in the subsequent lactation. These results show that high-yielding ewes do not have to experience metabolic disruption.

BCS in the peripartum period and DPL also affect milk yield and are associated with metabolic disorders [16,33,40]. A DPL shorter than 30 days and longer than 91–120 days reduced milk production in Lacaune dairy ewes [18]. Our ewes had an adequate DPL, and BCS and metabolite levels after lambing were all within normal physiological ranges, even when taking productivity into account. This observation may explain why our high-yielding ewes did not show metabolic disruptions. 

Lamb birth weight was positively associated with subsequent milk yield. These results are similar to those of a previous study that found that dairy cows with high birth weight calves (>40 kg) showed a greater increase in milk production at 305 days than cows with lighter calves (20 or 25 kg) [41]. This may be due to a natural predisposition to give more milk in order to meet the demands of bigger offspring. However, maternal BCS and weight in our study confound the observed relationship between lamb birth weight and subsequent milk yield: larger ewes with higher BCS produced more and gave birth to larger lambs. Male lambs were also generally bigger than female lambs. Unfortunately, our current study design does not allow us to control for these effects. We observed that ewes with male lambs had slightly higher daily milk yield. In contrast, a previous study found that ewes with female or larger lambs produced more milk than ewes with male lambs [21]. However, another study in dairy ewes reported that this effect did not occur [42]. The precise mechanisms by which fetal sex influences mammary gland development remains unknown [43]. 

In our study, we found that ewes bearing more than one lamb produced more milk than those bearing singletons, which is in agreement with previous studies showing that meat and dairy ewes with more than one offspring produce 3.4–9% more milk and have a lactation length 1% longer than ewes with singletons [19,21,42,44]. 

## 5. Conclusions

High-yielding Lacaune dairy ewes are better able to meet the metabolic demands of pregnancy and high milk production without showing signs of metabolic stress. As a result, they are able to maintain adequate BW and BCS throughout pregnancy and after lambing, without compromising productivity or fetal development. The highest-yielding animals produce lambs with the highest growth gains and continue to produce high yields in the subsequent lactation. Therefore, maternal productivity and lamb BW are not compromised by high milk yield in farms under the intensive management conditions of this farm [45]. 

## Figures and Tables

**Table 1:** Productivity parameters during concurrent gestation and lactation in mature ewes, and subsequent lactation in mature ewes and maiden sheep.

Lactation	Parameter	Mature Ewes (n = 224)	Maiden Sheep (n = 179)	All Sheep (n = 403)
Concurrent gestation dlactation	Lactation length, LL (days)	199.9 ± 56.7	N/A	N/A
	Total milk yield, TMY (L)	331.0 ± 140.9	N/A	N/A
	YDIMt (L/d)	1.65 ± 0.43	N/A	N/A
	YDIMb (L/d)	2.01 ± 0.65	N/A	N/A
	YDIMc (L/d)	1.19 ± 0.43	N/A	N/A
	YDIM in second month of pregnancy (L/d)	1.03 ± 0.42	N/A	N/A
	YDIM in third month of pregnancy (L/d)	0.93 ± 0.44	N/A	N/A
	YDIMd (L/d)	1.02 ± 0.40	N/A	N/A
	Dry period length, DPL (days)	80.3 ± 48.8	N/A	N/A
Subsequent	Lactation length, LLs (days)	200.1 ± 55.5 ^c^	217.0 ± 69.9 ^d^	207.8 ± 63.0
	Total milk yield, TMYs (L)	373.6 ± 171.6	348.5 ± 144.9	362.1 ± 160.2
	YDIMs (L/d)	2.13 ± 0.72 ^a^	1.85 ± 0.58 ^b^	2.00 ± 0.67
	YDIMts (L/d)	1.81 ± 0.61 ^a^	1.60 ± 0.51 ^b^	1.72 ± 0.57
	Dry period length, DLPs (days)	57.6 ± 20.0 ^c^	52.8 ± 14.1 ^d^	55.3 ± 7.43

YDIMb, Yield per day in milk before conception; YDIMc, Yield per day in milk during the month of conception; YDIMd, Yield per day in milk from conception to drying off; YDIMs, Yield per day in milk during first 6 months of lactation; YDIMt, Yield per day in milk during the whole lactation; YDIMts, Yield per day in milk during the whole lactation; N/A, not applicable. Data are expressed as mean ± standard deviation (S.D.). Superscripts indicate statistical significance between mature ewes and maiden sheep (a ≠ b *p* < 0.0001; c ≠ d *p* < 0.01).

**Table 2:** Productive parameters of ewes categorized by level of milk yield during the studied lactation and subsequent lactation.

Productivity Category	Values (Mean ± S.D.) (n)			
	TMY, L *	YDIMd, L/d *	YDIMc, L/d *	YDIMs, L/d **
Low yielding	161.0 ± 41.6 (56)	0.55 ± 0.15 (54)	0.65 ± 0.18 (56)	1.17 ± 0.31 (102)
Average yielding	301.5 ± 35.0 (89)	0.93 ± 0.10 (90)	1.13 ± 0.13 (89)	1.90 ± 0.18 (156)
High yielding	485.7 ± 91.2 (79)	1.42 ± 0.29 (80)	1.66 ± 0.25 (79)	2.70 ± 0.43 (145)
Total	331.0 ± 140.86 (224)	1.02 ± 0.39 (224)	1.19 ± 0.43 (224)	1.99 ± 0.67 (403)

TMY, Total milk yield over the lactation period; YDIMd, Yield per day in milk from conception to drying off; YDIMc, Yield per day in milk during the month of conception; YDIMs, Yield per day in milk during first 6 months of lactation. * Only mature ewes, categorized based on yield during the studied lactation. ** Mature and maiden sheep, categorized based on yield during the subsequent lactation.

**Table 3:** Phenotypic parameters and plasma metabolite levels in lambs born to mature ewes showing high, average, or low milk yield during concurrent gestation and lactation.

Parameters	Total Milk Yield			Yield Per Day in Milk from Conception to Drying off			Yield Per Day in Milk during the Month of Conception		
	L-TMY	A-TMY	H-TMY	L-YDIMd	A-YDIMd	H-YDIMd	L-YDIMc	A-YDIMc	H-YDIMc
**Phenotypic Parameters**	(*n* = 78)	(*n* = 118)	(*n* = 121)	(*n* = 82)	(*n* = 119)	(*n* = 116)	(*n* = 81)	(*n* = 122)	(*n* = 114)
Birth weight, kg	4.41 ± 0.89 ^j^	4.09 ± 0.87 ^k^	4.29 ± 0.74 ^j,k^	4.08 ± 0.86	4.31 ± 0.79	4.31 ± 0.84	4.11 ± 0.89 ^j^	4.20 ± 0.78 ^j,k^	4.40 ± 0.84 ^k^
Body weight, 17 days *, kg	7.40 ± 1.68 ^a^	8.88 ± 2.04 ^b^	9.63 ± 1.80 ^c^	8.68 ± 2.22 ^f,g^	8.46 ± 2.09 ^f^	9.23 ± 1.82 ^g^	8.65 ± 2.27	8.87 ± 1.95	8.83 ± 2.01
Average daily weight gain, kg	0.228 ± 0.1 ^k^	0.246 ± 0.07 ^j,k^	0.253 ± 0.06 ^j^	0.243 ± 0.07	0.240 ± 0.09	0.250 ± 0.07	0.238 ± 0.08	0.253 ± 0.08	0.239 ± 0.07
Birth trunk length, cm	30.10 ± 2.26	29.68 ± 2.02	30.05 ± 1.90	29.19 ± 2.35	30.30 ± 1.81	30.06 ± 1.91	29.35 ± 2.32 ^j^	30.04 ± 1.93 ^j,k^	30.21 ± 1.87 ^k^
Trunk length 17 days *, cm	36.93 ± 3.42 ^a^	39.36 ± 3.43 ^b^	40.44 ± 3.24 ^c^	38.63 ± 3.92 ^f^	38.97 ± 3.55 ^f,g^	39.76 ± 3.40 ^g^	38.54 ± 3.86	39.73 ± 3.31	39.03 ± 3.69
Birth BMI, kg/m^2^	48.45 ± 7.71	46.05 ± 6.76	47.39 ± 6.40	47.60 ± 8.05	46.65 ± 6.20	47.34 ± 6.76	47.23 ± 6.85	46.37 ± 6.96	47.92 ± 6.88
BMI, 17 days *, kg/m^2^	54.28 ± 10.52 ^f^	56.91 ± 9.32 ^f,g^	58.72 ± 9.04 ^g^	57.81 ± 10.86 ^j,k^	55.10 ± 8.87 ^j^	58.25 ± 9.28 ^k^	57.74 ± 10.75	55.88 ± 9.27	57.55 ± 9.19
Thoracic girth, 17 days *, cm	43.31 ± 3.56 ^a^	45.76 ± 3.80 ^b^	47.42 ± 3.51 ^c^	45.62 ± 4.18 ^f,g^	45.04 ± 3.95 ^f^	46.69 ± 3.65 ^g^	45.59 ± 4.30	45.69 ± 3.76	46.08 ± 3.93
Abdominal girth, 17 days *, cm	42.92 ± 4.03 ^a^	46.18 ± 4.43 ^b^	47.43 ± 4.51 ^b^	45.72 ± 4.66 ^j,k^	45.19 ± 4.62 ^j^	46.64 ± 4.72 ^k^	45.39 ± 4.78	46.04 ± 4.33	46.02 ± 5.02
**Metabolite Levels at 17 Days of age**									
Glucose, mg/dL	120.9 ± 20.0	120.1 ± 25.9	123.4 ± 26.6	117.3 ± 20.5	123.9 ± 27.0	122.2 ± 25.1	117.6 ± 25.1 ^j^	124.2 ± 25.8 ^k^	121.6 ± 23.3 ^k^
Lactate, mg/dL	19.7 ± 4.61 ^a^	19.7 ± 5.88 ^a^	17.4 ± 4.88 ^b^	19.4 ± 5.45 ^h^	19.5 ± 5.44 ^h^	17.7 ± 4.92 ^i^	19.7 ± 5.41	18.5 ± 5.41	18.5 ± 5.11
Cholesterol, mg/dL	91.4 ± 19.15	94.5 ± 16.43	95.6 ± 18.79	93.3 ± 17.18	94.0 ± 18.24	94.9 ± 18.54	94.6 ± 16.97	96.1 ± 19.21	91.7 ± 17.37
Triglycerides, mg/dL	51.3 ± 29.14	57.5 ± 32.30	54.2 ± 36.46	55.6 ± 42.69	52.9 ± 27.41	56.0 ± 31.02	57.5 ± 41.97	52.5 ± 26.88	55.1 ± 32.24
β-OHB, mmol/L	0.116 ± 0.08 ^d^	0.129 ± 0.06 ^d,e^	0.145 ± 0.07 ^e^	0.121 ± 0.07 ^k^	0.136 ± 0.07 ^j^	0.136 ± 0.05 ^j^	0.125 ± 0.07	0.143 ± 0.07	0.126 ± 0.06
NEFA, mmol/L	0.537 ± 0.15	0.546 ± 0.20	0.541 ± 0.16	0.517 ± 0.16	0.530 ± 0.15	0.571 ± 0.19	0.523 ± 0.17	0.547 ± 0.16	0.550 ± 0.19
Urea, mg/dL	31.1 ± 7.92 ^d^	33.9 ± 11.78 ^d^	35.7 ± 8.78 ^e^	33.7 ± 11.58	33.1 ± 10.25	34.9 ± 8.30	33.2 ± 12.06	34.1 ± 9.57	34.3 ± 8.69

* Lamb age at the second measurement was 17 ± 5 days. BMI, Body mass index; β-OHB, β-hydroxybutyrate; NEFA, non-esterified fatty acids; TMY, Total milk yield; YDIMd, Yield per day in milk from conception to drying off; YDIMc, Yield per day in milk during the month of conception. Data are mean ± S.D. a, b and c *p* < 0.0001; d ≠ e, *p* < 0.001; f ≠ g, *p* < 0.005; h ≠ i, *p* < 0.01; j ≠ k *p* < 0.05.

**Table 4:** Productivity parameters during the subsequent lactation in mature ewes and maiden sheep with single or multiple pregnancies.

Parameters	Age				Pregnancy Rank			
	**Mature Ewes** (*n* = 218)		**Maiden Sheep** (*n* = 179)		**Single** (*n* = 186)		**Multiple** (*n* = 211)	
LLs, days	200.1 ± 55.5 ^e^		217.0 ± 69.9 ^f^		208.6 ± 62.5		207.1 ± 64.7	
TMYs, L	373.6 ± 171.6		348.5 ± 144.9		355.5 ± 154.7		369.0 ± 167.4	
YDIMs, L/d	2.13 ± 0.72 ^a^		1.85 ± 0.58 ^b^		1.93 ± 0.63 ^g^		2.07 ± 0.71 ^h^	
YDIMts, L/d	1.81 ± 0.61 ^a^		1.60 ± 0.51 ^b^		1.68 ± 0.55		1.76 ± 0.60	
DLPs, days	57.6 ± 20.0 ^g^		52.8 ± 14.1 ^h^		54.1 ± 12.4		56.3 ± 21.2	
	**Single Pregnancy** **(*n* = 86)**	**Multiple Pregnancy** **(*n* = 132)**	**Single Pregnancy** **(*n* = 100)**	**Multiple Pregnancy** **(*n* = 79)**	**Mature Ewes** **(*n* = 86)**	**Maiden Sheep** **(*n* = 100)**	**Mature Ewes** **(*n* = 132)**	**Maiden Sheep** **(*n* = 79)**
LLs, days	196.1 ± 49.2	202.3 ± 59.6	219.4 ± 70.5	215.1 ± 72.0	196.1 ± 49.2 ^g^	219.4 ± 70.5 ^h^	202.3 ± 59.6	215.1 ± 72.0
TMYs, L	355.0 ± 158.6	386.9 ± 180.7	355.9 ± 152.1	339.1 ± 138.5	355.0 ± 158.6	355.9 ± 152.1	386.9 ± 180.7 ^g^	339.1 ± 138.5 ^h^
YDIMs, L/d	2.00 ± 0.66 ^g^	2.22 ± 0.75 ^h^	1.87 ± 0.59	1.82 ± 0.57	2.00 ± 0.66	1.87 ± 0.59	2.22 ± 0.75 ^c^	1.82 ± 0.57 ^d^
YDIMts, L/d	1.75 ± 0.56	1.86 ± 0.64	1.61 ± 0.53	1.59 ± 0.49	1.75 ± 0.56	1.61 ± 0.53	1.86 ± 0.64 ^c^	1.59 ± 0.49 ^d^
DLPs, days	56.4 ± 13.5	58.6 ± 23.5	52.3 ± 11.3	53.0 ± 17.0	56.4 ± 13.5 ^g^	52.3 ± 11.3 ^h^	58.6 ± 23.5 ^g^	53.0 ± 17.0 ^h^

DLPs, Dry period length; LLs, lactation length; TMYs, Total milk yield; YDIMs, Yield per day in milk during the first 6 months of lactation; YDIMts, Yield per day in milk during the whole lactation. Data are mean ± S.D. a ≠ b, *p* < 0.0001; c ≠ d, *p* < 0.001; e ≠ f, *p* < 0.01; g ≠ h, *p* < 0.05.

**Table 5:** Phenotypes of lambs born to high-, average-, or low-yielding ewes.

Phenotypic Parameters	L-YDIMs (*n* = 124)	A-YDIMs (*n* = 201)	H-YDIMs (*n* = 200)
Birth weight, kg	3.81 ± 0.92 ^d^	3.92 ± 0.96 ^de^	4.10 ± 0.85 ^e^
Body weight, 17 days *, kg	7.40 ± 2.0 ^a^	7.79 ± 2.0 ^a^	8.62 ± 2.2 ^b^
Average daily weight gain, kg	0.228 ± 0.007	0.229 ± 0.005	0.238 ± 0.005
Birth trunk length, cm	28.93 ± 2.50 ^f^	29.01 ± 2.61 ^f,g^	29.67 ± 2.04 ^g^
Trunk length 17 days *, cm	35.80 ± 3.96 ^a^	37.32 ± 3.85 ^b^	38.79 ± 3.66 ^c^
Birth BMI, kg/m^2^	44.92 ± 7.11 ^h^	45.91 ± 6.67 ^hi^	46.22 ± 6.68 ^i^
BMI, 17 days *, kg/m^2^	57.31 ± 10.45	55.45 ± 10.08	56.73 ± 10.15
Thoracic girth, 17 days *, cm	43.35 ± 3.92 ^a^	43.93 ± 4.24 ^a^	45.38 ± 4.15 ^b^
Abdominal girth, 17 days *, cm	42.89 ± 4.47 ^a^	43.85 ± 8.45 ^a^	45.60 ± 4.92 ^b^

* Lamb age at the second measurement was 17 ± 5 days. BMI: Body mass index. YDIMs, Yield per day in milk during the first 6 months of lactation. Data are mean ± S.D. a, b and c *p* < 0.0001; d ≠ e, *p* < 0.005; f ≠ g, *p* < 0.01; h ≠ i, *p* < 0.05.

**Table 6:** Productivity parameters in mature ewes and maiden sheep with male and/or female offspring during the lactation after having lambed those lambs.

Parameters	Male	Female	Male + Female	Female + Female	Male + Male
	(*n* = 95)	(*n* = 91)	(*n* = 111)	(*n* = 58)	(*n* = 39)
LLs, days	206.7 ± 57.9	210.6 ± 67.3	211.7 ± 68.7	201.8 ± 66.2	201.7 ± 48.9
TMYs, L	367.6 ± 165.0	342.7 ± 143.0	362.6 ± 167.9	394.5 ± 190.1	349.2 ± 123.8
YDIMs, L/d	2.02 ± 0.68 ^a^	1.84 ± 0.56 ^b^	2.02 ± 0.69	2.22 ± 0.80	1.99 ± 0.60
YDIMts, L/d	1.73 ± 0.58	1.62 ± 0.51	1.68 ± 0.60	1.91 ± 0.60	1.75 ± 0.57
DLPs, days	54.5 ± 13.2	53.7 ± 11.7	58.9 ± 26.7	53.5 ± 9.8	54.4 ± 18.5

DLPs, Dry period length; LLs, lactation length; TMYs, Total milk yield; YDIMs, Yield per day in milk during the first 6 months of lactation; YDIMts, Yield per day in milk during the whole lactation. Data are expressed as mean ± S.D. a ≠ b, *p* < 0.05.

**Table 7:** Productive parameters in mature ewes and maiden sheep with male and/or female offspring during subsequent lactation.

Parameters	Mature Ewes		Maiden Sheep		Mature Ewes			Maiden Sheep		
	Male (40)	Female (46)	Male (55)	Female (45)	Male + Female (73)	Female + Female (36)	Male + Male (21)	Male + Female (38)	Female + Female (22)	Male + Male (18)
LLs, days	207.0 ± 54.5	186.6 ± 42.5	206.5 ± 60.7 ^a^	235.2 ± 78.7 ^b^	200.5 ± 63.3	207.7 ± 63.0	199.1 ± 38.4	233.2 ± 74.4	192.2 ± 71.7	204.7 ± 59.9
TMYs, L	386.5 ± 174.3	327.6 ± 139.8	353.9 ± 158.1	358.3 ± 146.2	367.0 ± 180.7	443.8 ± 192.0	358.8 ± 143.0	354.2 ± 141.9	313.8 ± 160.4	338.1 ± 99.7
YDIMs, L/d	2.10 ± 0.73	1.91 ± 0.59	1.96 ± 0.64	1.76 ± 0.52	2.13 ± 0.72	2.46 ± 0.77	2.11 ± 0.75	1.80 ± 0.58	1.82 ± 0.72	1.85 ± 0.31
YDIMts, L/d	1.82 ± 0.58	1.69 ± 0.55	1.67 ± 0.57	1.54 ± 0.46	1.75 ± 0.63 ^a^	2.10 ± 0.56 ^b^	1.82 ± 0.69 ^a,b^	1.54 ± 0.52	1.60 ± 0.53	1.67 ± 0.38
DLPs, days	58.6 ± 15.9	54.3 ± 10.2	51.5 ± 9.8	53.3 ± 12.8	62.3 ± 31.3	54.7 ± 9.65	55.2 ± 11.4	53.7 ± 16.7	51.5 ± 10.0	53.6 ± 23.8

DLPs, Dry period length; LLs, lactation length; TMYs, Total milk yield; YDIMs, Yield per day in milk during the first 6 months of lactation; YDIMts, Yield per day in milk during the whole lactation. Data are expressed as mean ± S.D. a ≠ b, *p* < 0.05.

## Supplementary Materials

Tables S1–S3 are available online at https://www.mdpi.com/2076-2615/9/6/295/s1, Table S1: Estimates of Pearson correlation coefficients between lamb birth weight and maternal milk yield in mature ewes stratified by single vs. multiple pregnancies and by BCS score at different stages of pregnancy, Table S2: Estimates of Pearson correlation coefficients between lamb birth weight and maternal metabolite levels at mid-pregnancy (above) or late pregnancy (below) in mature ewes showing low, average, or high milk yield, Table S3: Estimates of Pearson correlation coefficients between milk yield during concurrent gestation and lactation and milk yield during the subsequent lactation in mature ewes with single or multiple pregnancies.
