# Supplementary material for: Maternal Metabolic Demands Caused by Pregnancy and Lactation: Association with Productivity and Offspring Phenotype in High-Yielding Dairy Ewes
Source: Animals (Basel). 2019 May 30;9(6):295. doi: 10.3390/ani9060295 (PMC6617180; doi:10.3390/ani9060295)
Supplement: Supplementary file 1 [file animals-09-00295-s001.pdf]

animals

Table S1. Estimates of Pearson correlation coefficients between lamb birth weight and maternal milk yield in mature ewes stratified by single *vs.* multiple pregnancies and by BCS score at different stages of pregnancy.

		Stage of pregnancy of mature ewes			
**Lambs born to: ↓**	**n**	**Month of conception (YDIMc)**	**Second month of pregnancy**	**Third month of pregnancy**	**Conception to drying off (YDIMd)**
All mature ewes	340	0.115 *	N/S	N/S	N/S
Single pregnancy	83	N/S	N/S	N/S	N/S
Multiple pregnancy	257	0.198 ****	N/S	N/S	0.128 *
**Maternal BCS at mid-pregnancy †**					
Thin (BCS ≤2)	68	N/S	N/S	N/S	N/S
Average (3>BCS>2)	180	N/S	N/S	N/S	N/S
Fat-BCS≥3	92	0.290 ***	0.277 **	0.313 ***	0.337 ****
**Maternal BCS at mid-pregnancy ‡**					
Thin (BCS ≤2)	26	N/S	N/S	N/S	N/S
Average (3>BCS>2)	133	0.226 **	N/S	N/S	0.175 *
Fat-BCS≥3	181	N/S	N/S	N/S	N/S

† 75 ± 5 d of pregnancy
‡ 142 ± 4 d of pregnancy
BCS, body condition score; YDIMc, Yield per day in milk during the month of conception; YDIMd, Yield per day in milk from conception to drying off; N/S, not significant.
**** $p < 0.001$; *** $p < 0.005$; ** $p < 0.01$; * $p < 0.05$

Table S2. Estimates of Pearson correlation coefficients between lamb birth weight and maternal metabolite levels at mid-pregnancy (above) or late pregnancy (below) in mature ewes showing low, average, or high milk yield.

		Mid-pregnancy (75 ± 5d of pregnancy)						
**Maternal milk yield ↓**	**n**	**GLU**	**LAC**	**CHO**	**TGL**	**β-OHB**	**NEFA**	**UR**
L-TMY	80	-0.192 †	N/S	N/S	N/S	N/S	N/S	N/S
A-TMY	129	0.176 *	N/S	N/S	N/S	N/S	N/S	N/S
H-TMY	129	N/S	0.160 †	0.182 *	-0.176 *	N/S	N/S	N/S
L-YDIMd	79	N/S	0.240 *	N/S	0.245 *	N/S	N/S	N/S
A-YDIMd	136	N/S	N/S	N/S	N/S	N/S	N/S	N/S
H-YDIMd	123	N/S	N/S	N/S	N/S	N/S	N/S	N/S
L-YDIMc	89	N/S	N/S	N/S	0.202 †	N/S	N/S	N/S
A-YDIMc	130	N/S	N/S	N/S	N/S	N/S	N/S	N/S
H-YDIMc	119	N/S	N/S	N/S	N/S	N/S	0.233 **	N/S
**Maternal milk yield ↓**		**Late-pregnancy (142 ± 4d of pregnancy)**						
L-TMY	80	-0.292 **	N/S	N/S	N/S	N/S	N/S	N/S
A-TMY	129	0.215 **	N/S	N/S	N/S	-0.200 *	-0.163 †	N/S
H-TMY	129	N/S	N/S	N/S	N/S	-0.250 **	-0.214 *	N/S
L-YDIMd	79	N/S	N/S	N/S	N/S	-0.200 †	N/S	N/S
A-YDIMd	136	N/S	-0.191 *	N/S	N/S	-0.146 †	-0.248 **	N/S
H-YDIMd	123	N/S	N/S	0.173 *	N/S	-0.299 ***	N/S	N/S
L-YDIMc	89	0.238 *	N/S	0.220 *	N/S	-0.248 *	-0.181 †	N/S
A-YDIMc	130	N/S	N/S	N/S	N/S	N/S	-0.187 *	N/S
H-YDIMc	119	N/S	N/S	N/S	N/S	N/S	N/S	N/S

β-OHB, β-hydroxybutyrate; CHO, Cholesterol; GLU, Glucose; LAC, Lactate; NEFA, non-esterified fatty acids; TGL, Triglycerides; UR, Urea; TMY, Total milk yield; YDIMd, Yield per day in milk from conception to drying off; YDIMc, Yield per day in milk during the month of conception; N/S, not significant. *** $p < 0.005$; ** $p < 0.01$; * $p < 0.05$; † $0.09 < p > 0.05$.

Table S3. Estimates of Pearson correlation coefficients between milk yield during concurrent gestation and lactation and milk yield during the subsequent lactation in mature ewes with single or multiple pregnancies.

		n	*r*
**Pregnancy rank**			
Single		86	0.662*
Multiple		132	0.604*
**Gender of offspring**			
Single	Male	40	0.705*
	Female	46	0.599*
Multiple	Male + Female	75	0.581*
	Female + Female	36	0.596*
	Male + Male	21	0.638*

* $p < 0.0001$

© 2019 by the authors. Submitted for possible open access publication under the terms and conditions of the Creative Commons Attribution (CC BY) license (http://creativecommons.org/licenses/by/4.0/).
